# Causes of Exocrine Pancreatic Insufficiency Other Than Chronic Pancreatitis

**DOI:** 10.3390/jcm10245779

**Published:** 2021-12-10

**Authors:** Lumír Kunovský, Petr Dítě, Petr Jabandžiev, Michal Eid, Karolina Poredská, Jitka Vaculová, Dana Sochorová, Pavel Janeček, Pavla Tesaříková, Martin Blaho, Jan Trna, Jan Hlavsa, Zdeněk Kala

**Affiliations:** 1 Department of Gastroenterology and Internal Medicine, University Hospital Brno, Faculty of Medicine, Masaryk University, 62500 Brno, Czech Republic; kunovsky.lumir@fnbrno.cz (L.K.); pdite.epc@gmail.com (P.D.); poredska.karolina@fnbrno.cz (K.P.); vaculova.jitka@fnbrno.cz (J.V.); 2 Department of Surgery, University Hospital Brno, Faculty of Medicine, Masaryk University, 62500 Brno, Czech Republic; sochorova.dana@fnbrno.cz (D.S.); janecek.pavel@fnbrno.cz (P.J.); kala.zdenek@fnbrno.cz (Z.K.); 3 Department of Gastroenterology and Internal Medicine, University Hospital Ostrava, Faculty of Medicine, University of Ostrava, 70852 Ostrava, Czech Republic; martin.blaho@fno.cz; 4 Department of Pediatrics, University Hospital Brno, Faculty of Medicine, Masaryk University, 61300 Brno, Czech Republic; jabandziev.petr@fnbrno.cz; 5 Central European Institute of Technology, Masaryk University, 62500 Brno, Czech Republic; 6 Department of Hematology, Oncology and Internal Medicine, University Hospital Brno, Faculty of Medicine, Masaryk University, 62500 Brno, Czech Republic; eid.michal@fnbrno.cz; 7 Department of Internal Medicine, Hospital Boskovice, 68001 Boskovice, Czech Republic; tesarikova.pavla@gmail.com; 8 Department of Gastroenterology and Digestive Endoscopy, Masaryk Memorial Cancer Institute Brno, 60200 Brno, Czech Republic

**Keywords:** exocrine pancreatic insufficiency, pancreatic enzyme replacement therapy, pancreatic cancer, cystic fibrosis, pancreatic resection, surgery, diabetes, celiac disease, inflammatory bowel disease, microbiome

## Abstract

Exocrine pancreatic insufficiency (EPI), an important cause of maldigestion and malnutrition, results from primary pancreatic disease or is secondary to impaired exocrine pancreatic function. Although chronic pancreatitis is the most common cause of EPI, several additional causes exist. These include pancreatic tumors, pancreatic resection procedures, and cystic fibrosis. Other diseases and conditions, such as diabetes mellitus, celiac disease, inflammatory bowel disease, and advanced patient age, have also been shown to be associated with EPI, but the exact etiology of EPI has not been clearly elucidated in these cases. The causes of EPI can be divided into loss of pancreatic parenchyma, inhibition or inactivation of pancreatic secretion, and postcibal pancreatic asynchrony. Pancreatic enzyme replacement therapy (PERT) is indicated for the conditions described above presenting with clinically clear steatorrhea, weight loss, or symptoms related to maldigestion and malabsorption. This review summarizes the current literature concerning those etiologies of EPI less common than chronic pancreatitis, the pathophysiology of the mechanisms of EPI associated with each diagnosis, and treatment recommendations.

## 1. Introduction

**Figure 1:**
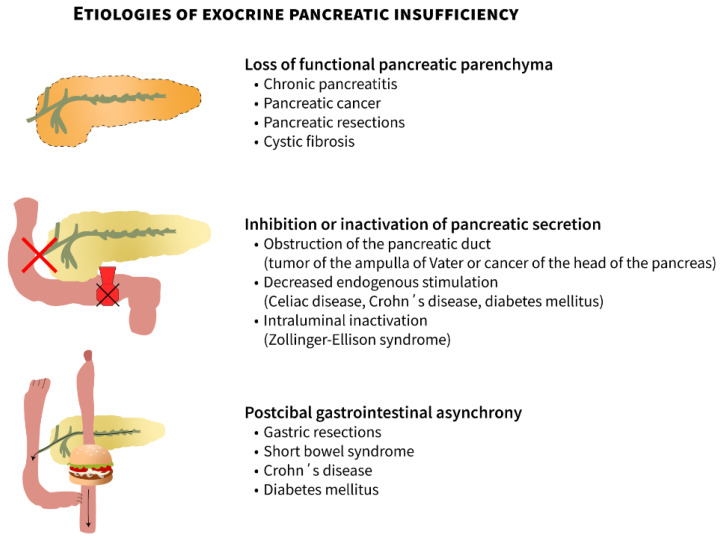
Etiologies of exocrine pancreatic insufficiency: (1) loss of pancreatic parenchyma, (2) inhibition or inactivation of pancreatic secretion, and (3) postcibal pancreatic asynchrony (edited according to [2] and created in collaboration with the Center for E-Learning Service at Masaryk University, Faculty of Informatics).

Exocrine pancreatic insufficiency (EPI), which is an important cause of maldigestion, malabsorption, and subsequently malnutrition, arises from primary pancreatic diseases or is secondary to impaired exocrine pancreatic function (either inhibition or inactivation of pancreatic secretion or postictal pancreatic asynchrony) [1]. The causes of EPI can be classified into three groups: (1) loss of pancreatic parenchyma, (2) inhibition or inactivation of pancreatic secretion, and (3) postcibal pancreatic asynchrony (Figure 1, adapted from [2]).

Furthermore, the etiology of EPI can be divided into diseases having obvious association with EPI (A) and diseases and conditions where the exact mechanisms responsible for EPI have not been elucidated (B) (Table 1, adapted from [1,3]).

Although the most common cause of EPI is chronic pancreatitis, several other causes also result in EPI. These include pancreatic tumors, pancreas resection, and cystic fibrosis (CF). Other diseases and conditions, such as diabetes, celiac disease, inflammatory bowel diseases, and advanced patient age, also have been shown to be associated with EPI, but the exact etiology of EPI has not been clearly elucidated in these situations. Pancreatic enzyme replacement therapy (PERT) is indicated for all conditions with clinically clear steatorrhea, weight loss, or symptoms related to maldigestion and malabsorption [4,5,6]. PERT should be administered to all patients with clinical symptoms of EPI or malabsorption in accordance with the guidelines for chronic pancreatitis [5,6].

## 2. Conditions Associated with EPI

### 2.1. Pancreatic Adenocarcinoma and Other Malignant Pancreatic Diseases

**Figure 2:**
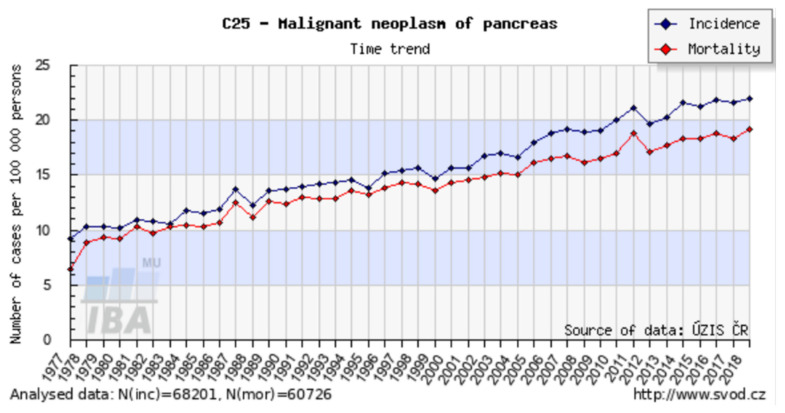
Incidence and mortality of pancreatic cancer in the Czech Republic. Available online: http://www.svod.cz (accessed on 19 September 2021).

In the Czech Republic, the incidence of pancreatic cancer is 20 per 100,000, and there are more than 2000 new cases annually. This incidence is the highest in Europe. Unfortunately, the number of patients with newly diagnosed pancreatic cancer continues to grow globally. The mortality curve follows the incidence curve (Figure 2), and pancreatic cancer has the lowest survival rate of all cancers [7]. It is estimated that by 2030 pancreatic cancer will become the second leading cause of cancer death. About 90% of all pancreatic tumors are adenocarcinomas, while neuroendocrine tumors and lymphomas are less common.

EPI is a known complication of both benign and malignant pancreatic diseases, pancreatic resection, and post-surgical alteration of the foregut’s anatomy. It is defined as inadequate pancreatic enzyme activity for digestion caused by insufficient pancreatic enzyme production, insufficient activation, or impaired enzyme deactivation [8]. EPI has been associated with high morbidity and mortality secondary to malnutrition-related complications and increased risk of cardiovascular events [9].

EPI is commonly disregarded among patients with pancreatic cancer. The most important predictors are the location of the tumor in the pancreatic head, >90% destruction of normal tissue, a degree of ductal obstruction, and surgical loss of pancreatic tissue [2,10,11]. The available data indicate that EPI occurs in 46–100% of patients with resectable tumors. Similarly, a high prevalence of EPI has been demonstrated in patients with unresectable pancreatic cancer. This was confirmed by Perez et al., who detected EPI in 75% of cases utilizing a 72 h fecal fat test, as well as by Partelli et al., who demonstrated extreme EPI (fecal elastase-1 [FE-1] ≤ 20 μg/g fecal matter) in 25%, severe EPI (FE-1 20–100 μg/g) in 14%, and moderate EPI (FE-1 100–200 μg/g) in 11% of patients with advanced pancreatic cancer. Patients with FE-1 ≤ 20 μg/g had a poorer prognosis (median survival: 7 versus 11 months, *p* = 0.031). Lower FE-1 level was more frequently diagnosed in patients with pancreatic head cancer, jaundice, and clinical steatorrhea [12,13].

Several mechanisms of EPI have been described. Pancreatic atrophy secondary to tumor-induced obstruction of the pancreatic duct and pancreatic fibrosis can lead to preoperative EPI. Early studies by Di Magno et al. demonstrated lower trypsin, lipase, and bicarbonate secretion after stimulation with cholecystokinin (CCK) in 17 patients with non-resected pancreatic cancer. Postoperative EPI is caused by the loss of pancreatic exocrine tissue and postcibal asynchrony [14].

Clinical symptoms associated with EPI include steatorrhea (characterized by large-volume, foul-smelling stools), diarrhea, weight loss, flatulence, and abdominal pain. Fat and protein malabsorption correlate significantly with weight loss, the degree of which correlates with reduced survival [15,16]. Although patients with pancreatic ductal adenocarcinoma (PDAC) who receive PERT achieve increased median survival, the use of PERT among patients with PDAC appears low [17]. An indication for PERT, according to expert opinion, is progressive weight loss and steatorrhea, defined as at least 7–15 g fecal fat per day on a diet of 100 g fat per day [10,18]. To achieve optimal lipid digestion, 25,000–50,000 PhU of lipase are required for a typical meal. Only a few studies have addressed the utility of PERT for patients with inoperable pancreatic cancer. Dominguez-Munoz et al. presented a retrospective, nonrandomized case series of 76 patients with inoperable cancers. Patients received PERT with nutritional counseling and palliative care (n = 45) or standard palliative care without PERT (n = 21). The median survival of patients with PERT was longer than the survival of patients with standard palliative therapy alone (301 days versus 89 days) [19].

Furthermore, findings by Bruno et al. indicate that patients with unresectable pancreatic cancer could benefit from PERT to decelerate weight loss [20]. By contrast, in a double-blind, placebo-controlled study in patients with unresectable pancreatic cancer (43% had severe EPI), mean weight loss after 8 weeks in patients receiving PERT (−1.49%) was not significantly different from that in the placebo group (−2.99%). PERT did, however, improve the nutritional status in a subset of patients with unresectable pancreatic head region cancer [21]. Finally, in 2020, de Iglesia et al. published a systematic review and meta-analysis of 11 studies. The results indicate that PERT was associated with a survival benefit of 3.8 months (95% confidence interval: 1.37–6.19) [22].

Core tips: EPI occurs in a majority of patients with either resectable or unresectable pancreatic cancer and is more common in patients with pancreatic head cancer, jaundice, and clinical steatorrhea. EPI leads to fat and protein malabsorption and weight loss, the degrees of which correlate with reduced survival. The value of PERT in PDAC patients is generally underappreciated. Moreover, it leads to increased median survival also in patients with inoperable PDAC when compared with standard palliative therapy alone.

### 2.2. Pancreatic Resections

As mentioned in the previous section, pancreatic head carcinoma is a common reason for pancreatic resections. Pancreatic resections are performed for periampullary tumors (lesions of the pancreatic head and uncinate process, distal bile duct, and ampulla of Vater), duodenal tumors, mucinous lesions with malignant potential (such as intraductal papillary mucinous neoplasms and mucinous cystic neoplasms), neuroendocrine tumors, and chronic pancreatitis [23]. Types of pancreatic resections are pancreaticoduodenectomy (classical Whipple procedure), pylorus-preserving pancreaticoduodenectomy (ppWhipple), distal pancreatectomy, and total pancreatectomy [24]. Anatomical and hormonal changes after pancreatic surgery can cause EPI and maldigestion. The main causes that contribute to EPI are abnormal fundus relaxation, reduction of CCK production caused by duodenal resection (the classical Whipple operation), loss of pancreatic tissue, or gastropancreatic asynchrony [4,24]. Some studies suggest that the severity and prevalence of EPI differ if pancreaticogastrostomy (P-G) or pancreaticojejunostomy (P-J) is used in the reconstruction. A significant effect in such cases is low pH in the stomach, which inactivates pancreatic enzymes in the case of P-G. Jang et al. presented a study comparing P-G with P-J in postoperatively developed EPI. Although this was a study with a low number of patients, it found that 100% of patients with P-G developed severe EPI, while only 75% of the patients with P-J developed severe EPI and 25% mild EPI, suggesting that P-G has higher chance of resulting in severe EPI than does P-J [25,26].

EPI is an underestimated complication of pancreatic surgery, but one that can have significant impacts on patients’ quality of life. Weight loss, malnutrition, lack of absorption of fat-soluble vitamins, and steatorrhea are typical symptoms in the postoperative period and can shorten the life expectancy of patients. The severity of EPI depends upon preoperative exocrine function, primary diagnosis, type of resection, and volume of resected tissue. Yuasa et al. published a study in 2012 comparing EPI prevalence in patients after pancreatoduodenectomy and distal pancreatectomy and concluded that pancreatoduodenectomy induced EPI more frequently than did distal pancreatectomy [26,27].

In diagnosing EPI, the fecal elastase-1 test is one of the most commonly used tests in the clinical setting. It has high sensitivity in cases of moderate to severe EPI and there are no special requirements of the patient. In addition, PERT need not be discontinued before the test, because artificial enzymes do not cross-react with the human elastase antibody [24]. Another frequently used option is the ^13^C-mixed triglyceride breath test, although it has disadvantages similar to those of the fecal elastase-1 test inasmuch as it has limited sensitivity in mild cases of EPI [28].

In 2017, Roeyen et al. published a prospective study evaluating pancreatic function in 78 patients after pancreatoduodenectomy using the ^13^C-labeled mixed-triglyceride breath test preoperatively and postoperatively. The results revealed that 64.1% of the patients required postoperative enzyme replacement therapy [29]. In another study, Lim et al. observed exocrine and endocrine insufficiency in a prospective study with 227 patients who underwent pancreatic surgery. From 94 patients without preexisting exocrine insufficiency, EPI appeared postoperatively in all 94 cases [30].

The goal in treating EPI is to overcome clinical symptoms caused by enzyme deficiency. To reach this goal, dietary measures, a change in lifestyle, and PERT are necessary. Dietary recommendations call for small, frequent, high-energy meals. Fat restriction intake is no longer recommended because it exacerbates fat-soluble vitamin deficiency [31]. PERT remains a cornerstone of therapy. Dosage should be individualized for each patient, but a minimum dose of 40,000–50,000 USP units of lipase per main meal and 20,000–25,000 per snack should be prescribed. In some cases where PERT achieves an insufficient response, the addition of a proton pump inhibitor (PPI) can help. By raising gastric pH, the PPI increases enzyme replacement so as to obtain the expected effect [26,32]. Screening for lower levels of fat-soluble vitamins should be considered in patients with EPI and replacement therapy should be introduced if a deficiency is found. However, there has been insufficient study of blanket fat-soluble vitamin replacement; therefore, a case-by-case clinical approach is advised [5].

Core tips: Several types of pancreatic resections are performed for both pancreatic and extrapancreatic indications. EPI is an underestimated complication of pancreatic surgery, but it can have significant impacts on patients’ quality of life and can shorten life expectancy. Pancreatoduodenectomy induces EPI more frequently than distal pancreatectomy. Pancreaticogastrostomy seems to bear a higher risk of EPI than pancreaticojejunostomy, probably because of low pH in the stomach, which inactivates pancreatic enzymes. PERT should become an integral part of therapy for patients after pancreatic resections.

### 2.3. Obstruction of the Pancreatic Duct

There are many possible causes for obstruction of the pancreatic duct, the main ones being pancreatic cancer and chronic pancreatitis. Less common etiologies of pancreatic duct obstruction include tumors of the distal bile duct or ampullary adenocarcinoma. Carcinoma of the ampulla of Vater is a less common tumor with an incidence of approximately 0.2% among all gastrointestinal cancers, and it has a better surgical resection rate and overall prognosis compared to pancreatic cancer [33,34]. Obstruction of the pancreatic duct impedes the transport of pancreatic enzymes into the duodenum. Increased pressure in the duct, upstream from the site of obstruction, may cause atrophy of the surrounding gland and hence also decreased enzyme production.

The work of Di Magno et al. comparing the length of the opacified duct obtained by retrograde pancreatography and secretory capacity of the gland showed that a decrease in exocrine pancreatic secretion cannot be detected until more than 60% of the main pancreatic duct’s total length has been obstructed [14].

Core tips: The obstruction of the pancreatic duct impedes the transport of pancreatic enzymes into the duodenum and increases pressure in the duct. This may cause atrophy of the surrounding gland and hence decrease enzyme production to a point of EPI.

### 2.4. Cystic Fibrosis

**Figure 3:**
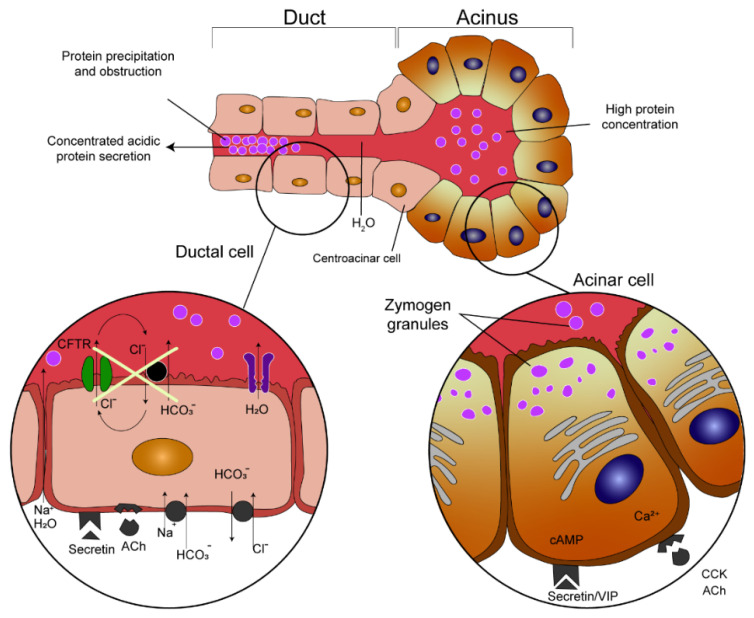
Pathogenesis of pancreatic duct obstruction in cystic fibrosis patients (edited according to [37,38] and created in collaboration with the Center for E−Learning, Faculty of Informatics at Masaryk University).

CF is a genetic defect with autosomal recessive inheritance caused by a mutation in the CF transmembrane conductance regulator (*CFTR*) gene [35]. The *CFTR* gene encodes a CFTR protein that functions primarily as a transmembrane chloride channel. It is also highly expressed in the pancreatic duct epithelia. Here it permits anions (bicarbonates in particular) and water to enter the ductal lumen and thus allows highly concentrated proteins secreted by the acinar cells to remain in a soluble state. Therefore, reduced or nonexistent CFTR channel function results in reduced volumes of pancreatic juice and hyperconcentration of macromolecules. That leads to precipitation in the lumen of the ducts, causing them to become obstructed and damaged [36] (Figure 3, edited according to [37,38]).

**Figure 4:**
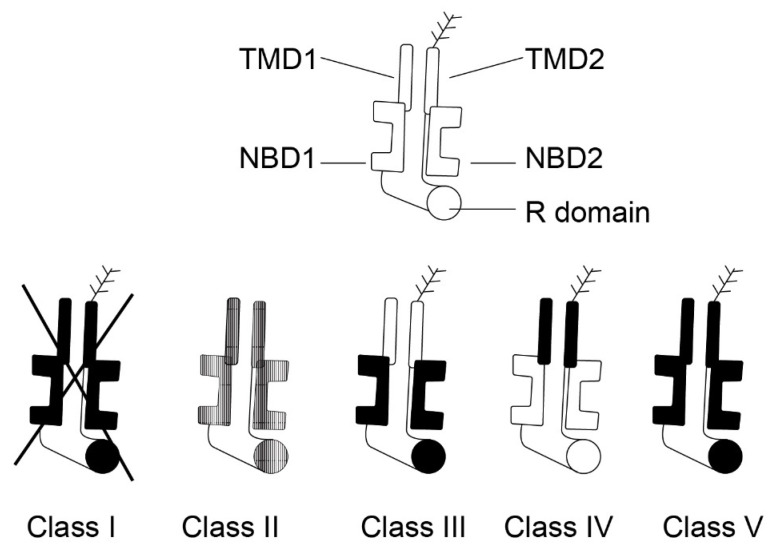
Five classes of *CFTR* mutations (edited according to [41], created in collaboration with Service Center for E-Learning at Masaryk University, Faculty of Informatics).

The pancreas is one of the organs earliest and most affected by CF. Almost 90% of patients with CF present with EPI and *CFTR* gene mutations constitute the vast majority (about 85%) of children with pancreatic insufficiency. However, the extent of pancreatic disease varies. The clinical presentation of individual cases depends upon the combination of different *CFTR* mutations, the potential presence of modifier gene mutations, and environmental factors [39]. The *CFTR* mutations are historically divided into five classes according to the type of defect caused to the CFTR protein and also according to the severity of the clinical phenotype: severe mutations (classes 1–3) and mild mutations (classes 4–5) [40]. These are detailed in Figure 4 (edited according to [41]). In general, the functional consequences of *CFTR* mutations depend upon the combined effects of both *CFTR* alleles and the severity of the phenotype depends upon the mildness or severity of the mutations [42].

From a clinical perspective, patients with CF can be divided into those who are pancreatic sufficient (PS) and pancreatic insufficient (PI). While the majority of CF patients are PI, as many as 15% possess sufficient pancreatic exocrine function to permit normal digestion [43]. Recurrent acute pancreatitis or chronic pancreatitis develop in less than 2% of CF patients, first appearing in adolescence or young adulthood and almost exclusively among PS patients [44]. PI patients are usually free of these complications because functional acinar tissue has been lost in utero or soon after birth.

Delivery of active digestive enzymes into the proximal small intestine is a cornerstone in the treatment of EPI in CF patients [45]. Acid suppression tends to play a larger role because bicarbonate secretion (by the pancreas, duodenum, and biliary tree) is usually disrupted more than in other forms of CP [46]. Vitamin deficiencies may be present, and CF patients should be monitored and supplemented accordingly [47].

Several novel therapeutic agents have recently been developed to increase CFTR protein activity. We can now specifically target the gene defect and not only treat the resulting symptoms. Indication for the use of these agents depends upon the presence of a specific *CFTR* mutation [48]. Ivacaftor acts as a CFTR protein potentiator. It increases the probability of the CFTR channel opening and hence it facilitates chloride transport [49,50]. The lumacaftor acts as a CFTR protein corrector by assisting in its formation and increases the amount of functional CFTR protein on the cell surface [51]. Tezacaftor is the next molecule currently available to correct CFTR protein structure [52]. These therapeutic agents are now being tested and are used in combination [51,52].

Core tips: The pancreas is one of those organs earliest and most affected by CF. Almost 90% of patients with CF present with EPI. The extent of pancreatic disease varies, however, and the clinical presentation depends upon the combination of different *CFTR* mutations, the potential presence of modifier gene mutations, and environmental factors. Thus, patients with CF can be divided into those who are pancreatic sufficient (up to 15%) and pancreatic insufficient. Recurrent acute pancreatitis or chronic pancreatitis develops in less than 2% of CF patients and almost exclusively among pancreatic sufficient patients. PERT is a cornerstone in the treatment of EPI in CF patients, with acid suppression playing an important role because bicarbonate secretion is usually disrupted more than in other pancreatopathies. Several novel therapeutic agents (ivacaftor, lumacaftor, and tezacaftor) have been developed to increase CFTR protein activity.

### 2.5. Exocrine Pancreatic Insufficiency in Children

A variety of pancreatic diseases can be encountered in the pediatric population, despite that these diseases are not considered very common. The incidence of acute, acute recurrent, and chronic pancreatitis may be similar to that in adults. CF is the most common cause of EPI in children. Table 2 describes the other most common causes [53]. PERT is an integral part of treatment in pediatric patients with EPI, as otherwise, they may face malnutrition due to inadequate nutrient absorption and their growth may be adversely affected [54].

#### 2.5.1. Shwachman–Diamond Syndrome

Shwachman–Diamond syndrome (SDS) is a rare, autosomal, recessively inherited disorder. As many as 90% of children with SDS have mutations in the SBDS gene, which is located on chromosome 7q11. SDS is characterized by congenital anomalies, bone marrow defects, EPI, and short stature, but clinical manifestations can be heterogeneous and involve the skeletal, cardiovascular, nervous, gastrointestinal, renal, and other systems. The most common hematological abnormality is neutropenia in as many as 80% of patients. Patients with SDS tend to develop myelodysplasia and leukemia over time. Recurrent infections are common and are a frequent cause of death. Although only about one-half of patients report diarrhea, low levels of fecal elastase may be present at diagnosis in more than three-quarters of patients. EPI is often transient, however, and steatorrhea may improve spontaneously over time. Two-thirds of patients experience failure to thrive. Despite adequate PERT, poor growth usually does not improve. The diagnosis of SDS is often made on the basis of the clinical picture and on the demonstration of EPI and hematological abnormalities while excluding other causes of EPI and bone marrow failure. Consensus guidelines are available to facilitate early diagnosis and therapy [53,55,56,57].

Core tips: SDS is a rare autosomal recessive disorder characterized by congenital anomalies, bone marrow defects, EPI, and short stature. Typical presentation includes neutropenia (~80%), failure to thrive (~66%), diarrhea (~50%), and skeletal abnormalities (~33%). Poor growth usually does not improve despite adequate PERT. EPI is often transient, however, and steatorrhea may spontaneously improve over time. Recurrent infections are common and a common cause of death.

#### 2.5.2. Chronic Pancreatitis

The exact prevalence of EPI in pediatric patients with chronic pancreatitis (CP) is unknown, but according to available sources, approximately one-third of children with CP could be exocrine pancreatic insufficient [53,58].

#### 2.5.3. Johanson–Blizzard Syndrome

Johanson–Blizzard syndrome is a rare autosomal recessive disorder caused by mutations in the *UBR1* gene that lead to the destruction of pancreatic acini in utero. Typical clinical presentation includes EPI, multiple congenital anomalies, hypothyroidism, and developmental delay [53,59,60].

#### 2.5.4. Pearson Syndrome

Pearson syndrome is a rare disease resulting from sporadic mutations in mitochondrial DNA leading to a defect in oxidative phosphorylation. Patients usually present in infancy with severe hematological abnormalities (macrocytic anemia, neutropenia, and thrombocytopenia). Other characteristic symptoms include exocrine and endocrine pancreatic dysfunction, hyperlipidemia, hepatic steatosis, proximal renal tubular insufficiency, metabolic acidosis, and failure to thrive [53,61].

#### 2.5.5. Other Causes

Jeune syndrome, pancreatic hypoplasia and aplasia, and isolated pancreatic enzyme deficiencies are rare causes of EPI in childhood [53].

## 3. Poorly Clarified Mechanisms Associated with EPI

### 3.1. Diabetes Mellitus

The relationship between impaired exocrine pancreatic function and diabetes mellitus has been repeatedly described [62]. It has also been documented several times that approximately 50% of patients with type 1 diabetes mellitus show demonstrable exocrine pancreatic dysfunction when using direct stimulation tests, and exocrine dysfunction can also be identified in 30–50% of patients with insulin-dependent type 2 diabetes [63,64]. There can be no question today whether there is a relationship between the high prevalence of diabetes and pancreatic diseases such as chronic pancreatitis (including autoimmune pancreatitis) and nonalcoholic fatty pancreas disease (NAFPD) [65]. Metabolic syndrome and its components, obesity, dyslipidemia, and arterial hypertension are all etiological factors implicated in the development of acute and chronic pancreatitis, and in the development of NAFPD, a condition that has intrigued gastroenterologists worldwide. NAFPD may be associated with impaired glucose metabolism, lipotoxicity, insulin resistance, and inflammation. All these changes lead to changes in glucose metabolism. Smits et al. [66] defined pancreatic steatosis as a state characterized by the accumulation of fat in the pancreatic parenchyma of patients who demonstrably abstain from alcohol intake. A representative study published in 2013 confirmed the relationship between NAFPD and pancreatic beta cells [67]. NAFPD may therefore be associated with both the dysfunction and the number of beta cells in the pancreas.

All types of diabetes (i.e., diabetes mellitus type 1, diabetes mellitus type 2, and pancreatogenic diabetes mellitus [type 3c]) are associated with manifest exocrine pancreatic impairment [68]. Exocrine pancreatic insufficiency found in diabetes is usually of mild to moderate intensity and is not associated with steatorrhea. The prevalence of exocrine pancreatic dysfunction is higher in type 1 diabetes than in type 2, at 26–57% versus 20–36%, respectively [69]. As a rule, all patients suffering from pancreatogenic diabetes show exocrine pancreatic dysfunction. All patients with pancreatogenic diabetes should be thoroughly examined for possible changes in exocrine pancreatic secretion. In the event of any decrease or dysfunction in that secretion, the patients should be treated with standard pancreatic supplementation.

Abnormalities in exocrine pancreas secretion are associated with diabetes mellitus. Frier et al. [70] described a decrease in stimulated pancreatic secretion in some patients with diabetes mellitus. A decrease in amylase secretion was found in 66% and trypsin secretion in 44% of patients with type 1 diabetes, and the degree of these dysfunctions was positively correlated with the duration of diabetes. Similarly, the secretion of bicarbonate after appropriate stimulation was also significantly reduced. In patients with type 2 diabetes hyperglucagonemia was observed, and this was associated with inhibition of pancreatic enzyme production (of amylase, lipase, and trypsin) [71]. Increased levels of somatostatin, which have been well documented since the 1980s, are also accompanied by the inhibition of exocrine pancreatic secretion, especially of bicarbonate, but also of amylase and trypsin [72].

There exist several theories as to why there is a decrease in exocrine pancreatic secretion in patients with diabetes mellitus. One of the possible explanations involves pancreatic atrophy and fibrotization of the tissue, changes in the release of some gastrointestinal mediators, autonomic pancreatic neuropathy, and changes in the immune system [1]. In some patients with diabetes mellitus, there was a documented imbalance between insulin and glucagon, rather than a defect in somatostatin levels (stimulation versus inhibition) [62]. In diabetes patients, insulin deficiency leads to atrophy of the gland.

Regulation of pancreatic enzyme production and secretion depends upon the presence of gastrointestinal hormones and the existence of local neuronal signals. In as many as 50% of cases, postprandial exocrine pancreatic secretion is mediated by gastrointestinal tract peptides. These, however, can be affected by the presence of pancreatic neuropathy [73]. Diabetic microangiopathy can reduce pancreatic perfusion and be associated with fibrotization of the gland. Autoimmune diseases affect both exocrine and endocrine functions of the pancreas. Antibodies directed against the cells of the pancreatic islands also react with the acinar cells of the pancreas. As such, changes in exocrine and endocrine pancreatic secretion are characteristic of the presence of autoimmune disorders [74].

Pancreatogenic diabetes (type 3c) develops as a consequence of previous pancreatic disease, surgical resection procedures, or traumatic damage to the gland. Its prevalence among the diabetic population is estimated to be 5–10% [75,76].

The most common etiological factor in the development of type 3c diabetes is represented by chronic pancreatitis (76–79%). Pancreatic adenocarcinoma is implicated in 8–9% of cases, as is hemochromatosis. CF is responsible for this type of diabetes in 4% of patients, and 2–3% of cases are caused by surgical resection procedures [76]. It is an established fact that all patients diagnosed with type 3c diabetes also show signs of EPI [77].

Core tips: All types of diabetes are associated with exocrine pancreatic impairment. Exocrine pancreatic dysfunction is demonstrable in approximately 50% of patients with type 1 diabetes mellitus and in 30–50% of patients with insulin-dependent type 2 diabetes. As a rule, all patients suffering from pancreatogenic diabetes show exocrine pancreatic dysfunction. Furthermore, there is an undeniable relationship between the high prevalence of diabetes and pancreatic diseases such as chronic pancreatitis (including autoimmune pancreatitis) and nonalcoholic fatty pancreas disease (NAFPD) as a part of metabolic syndrome. Therefore, diabetics should be screened and treated for EPI.

### 3.2. Celiac Disease

Celiac disease is a chronic inflammatory disorder of the small intestine produced in susceptible people by ingestion of dietary gluten [78]. Globally, celiac disease’s prevalence is about 1% [79]. The disease is characterized by intestinal malabsorption associated with villous atrophy of the small intestinal mucosa, clinical and histological improvement after adherence to a strict gluten-free diet, and relapse when gluten is reintroduced. Presentation of celiac disease varies widely, ranging from asymptomatic forms to severe diarrhea, weight loss, and nutritional deficiencies [80]. Therapy for the disease consists of a lifelong gluten-free diet.

EPI is caused by reduced or inappropriate secretion or activity of pancreatic juice and its digestive enzymes, most especially pancreatic lipase. EPI can result in clinical manifestations such as steatorrhea, weight loss, and biochemical alterations related to malabsorption and maldigestion of lipids and liposoluble micronutrients [81].

EPI is associated with celiac disease and is a recognized cause of chronic diarrhea [82]. The prevalence of EPI ranges from 4% to 80% in patients with untreated celiac disease [1]. EPI’s pathophysiology in celiac disease appears to be multifactorial but is not fully understood [83]. Impaired secretion of pancreatic stimulating hormones from the atrophied proximal small intestine is regarded as the most likely mechanism for EPI [84]. The proteolytic enzyme fecal elastase-1, produced specifically by the pancreas, is useful in identifying exocrine pancreatic insufficiency in adult celiac patients with diarrhea [82]. Fecal elastase-1 is reported to have high specificity and sensitivity in patients with severe exocrine insufficiency, and it is commonly low (less than 200 μg/g stool) in patients with celiac disease and chronic diarrhea [85].

PERT is a cornerstone in the treatment of EPI. The goals of this treatment are to normalize digestion, alleviate EPI-related symptoms, prevent malnutrition-related morbidity, and avert any general progression toward mortality [3]. Patients with celiac disease on a gluten-free diet and with low fecal elastase levels should receive PERT [10].

Evans et al. presented a prospective case series of 20 patients with celiac disease, persistent chronic diarrhea, and EPI (diagnosed by FE-1 of less than 200 μg/g fecal matter). Of these 20 patients, 19 initially improved on PERT. The mean daily dose of pancreatin was 45,000 units of lipase. In the whole group, there was a significant increase in FE-1 levels over time. The median value was 90 μg/g at 0 month and 365 μg/g at follow-up (45–66 months). Furthermore, there was a significant long-term improvement in their chronic diarrhea, demonstrated by a reduction in the median frequency of stool production from 4 per day to 1 per day. However, no increase of weight was observed [82]. Conversely, Carroccio et al. presented a double-blind randomized trial of 40 children with celiac disease on a gluten-free diet and treated with PERT. Data from this trial demonstrated that PERT increased body weight versus placebo during the first 30 days after diagnosis [86]. Moreover, Leeds et al. presented results from a study of 20 adults with celiac disease showing that PERT reduced chronic diarrhea from four stools to one stool per day in 90% of patients. Pancreatic enzyme supplementation may therefore provide symptomatic benefits for those celiac patients with chronic diarrhea and low FE-1 [85].

Core tips: In patients with untreated celiac disease, the prevalence of EPI ranges from 4% to 80%. Impaired secretion of pancreatic stimulating hormones from the atrophied proximal small intestine is considered to be the most likely mechanism for EPI in celiac disease. Patients with celiac disease on a gluten-free diet with persistent diarrhea and low fecal elastase levels should receive PERT.

### 3.3. Inflammatory Bowel Diseases

Inflammatory bowel diseases (IBD) involving Crohn’s disease (CD) and ulcerative colitis (UC) are characterized by chronic relapsing inflammation of the gastrointestinal tract. The etiology of IBD is still not well understood, but it is presumed to involve genetics, immune response, as well as environmental and microbial factors [87,88]. Although IBD mainly affects the intestine, the involvement of other organs (extraintestinal manifestation, EIM) can occur. In addition to the most typical EIMs as arthritic, cutaneous and hepatobiliary, pancreatic disorders have been recorded in patients with IBD [89,90]. These pancreatic abnormalities include a heterogeneous group of conditions, such as acute, chronic, or autoimmune pancreatitis, EPI, enzyme elevations, or imaging abnormalities [91,92]. Furthermore, in patients without clinical symptoms of pancreatic disease, autopsy studies have shown a pancreatic abnormality in as many as 53% of UC patients and 38% of patients with CD [93,94].

The prevalence of EPI (based on low fecal elastase) in patients with IBD is reported to range from 18% to 80% of cases [90,95], keeping in mind the possibility for false positive patients diagnosed with EPI due to secondary low fecal elastase in cases of diarrhea. In contrast, EPI may have a very similar clinical presentation as do IBD and might therefore be underdiagnosed in these patients [90,93,96]. The diagnosis of EPI in IBD patients, therefore, remains a clinical challenge.

The causes of EPI in IBD patients are diverse. IBD patients are at increased risk of chronic pancreatitis, which can progress to fibrosis and destruction of the pancreatic parenchyma and result in EPI [6,97]. Chronic pancreatitis in IBD patients is mainly idiopathic, due to primary sclerosing cholangitis, autoimmune pancreatitis, or primary biliary cholangitis [6,98]. EPI without imaging abnormalities or elevated serum amylase levels is observed in up to 18% of IBD patients, and in some cases is reported as transient [88,93]. EPI might, in addition, be associated with disease activity in IBD patients, as recent studies indicate that EPI regresses in disease remission [93,99]. It is suggested that adequate treatment of IBD might normalize the level of the serum fecal elastase. This could be explained by an autoimmune origin of EPI and is described as an extraintestinal complication of IBD [93,100]. In terms of immune-mediated etiology of EPI, pancreatic autoantibodies (PABs) have been identified and occur in as many as 39% of CD patients, up to 23% of UC patients, but in only 3% of healthy controls according to most studies. The target of PABs is the exocrine pancreas, but the association between PABs and exocrine insufficiency or changes in the pancreatic duct has not been confirmed. Therefore, the occurrence of PAB in patients with IBD gives rise to diagnostic challenges [6,101].

In particular, the prevalence of EPI in CD patients varies between 14% and 30% [1]. According to some studies, the extent of bowel involvement, disease activity, and its localization appeared to be related to the intensity of exocrine pancreatic insufficiency [90,98]. The cause of EPI in CD patients remains ambiguous, as mentioned above. Possible mechanisms include malnutrition, PABs, pancreatic duct imaging abnormalities, and reduced pancreatic secretion [1,90]. PABs could explain an immunological induction of EPI in CD patients; however, the association remains unclear, as discussed earlier [1,6,101]. Secondly, pathological changes in the pancreatic duct obstructing the flow may also result in EPI. In CD patients, an inflamed ampulla of Vater or fistula formation in this region can lead to duodenal reflux into the pancreatic duct and consequent damage to the same [1,102]. Finally, intestinal inflammation and scarring can decrease hormone secretion through the intestinal wall, thus resulting in decreased activation of the pancreas and in EPI [1,90].

Whereas EPI is reported in as many as 22% UC patients, severe EPI (characterized as fecal elastase less than 100 μg/g) is reported in up to 9% of patients [1,24]. The etiology of EPI in UC patients is mainly associated with chronic pancreatitis, autoimmune pancreatitis, or pancreatic duct abnormalities. The latter has been found by magnetic resonance cholangiopancreatography in up to 16% of UC patients without a history of alcohol abuse or previous acute pancreatitis [103,104].

Despite many studies dealing with pancreatic abnormalities in IBD patients, none has evaluated the possible effects of PERT. To date, there have not been many published guidelines [1].

Although pancreatic insufficiency seems to be a rather frequently occurring EIM of IBD, its clinical significance remains undefined. Elucidating the etiology of EPI could improve the quality of life in patients with IBD in the future. However, the number of high-evidence level reports in this field is so far limited.

Core tips: Pancreatic insufficiency seems to be a rather frequently occurring extraintestinal manifestation of IBD. Nevertheless, its clinical significance remains undefined. The causes of EPI in IBD patients are diverse and can include increased risk of chronic pancreatitis, autoimmune-mediated process associated with IBD activity, inflammatory involvement of periampullary duodenum leading to obstruction and/or duodenal reflux into the pancreatic duct, and intestinal inflammation and scarring that decrease hormone secretion from the intestines. To date, however, there are no significant studies and/or guidelines addressing the possible effects of PERT in IBD patients.

### 3.4. Gastrointestinal Surgeries Other Than Pancreatic Resections

Upper gastrointestinal surgery can have a significant impact on the absorption and digestion of nutrients in the entire gastrointestinal tract (GIT). According to a study by Domínguez-Muñoz, approximately 80% of patients with surgical procedures in the upper GIT exhibit maldigestion, and EPI can contribute to any pathogenesis. We will focus on extrapancreatic gastrointestinal surgical procedures. Significant EPI has occurred in patients with gastric resections, small intestinal resections, and even esophageal resections.

**Figure 5:**
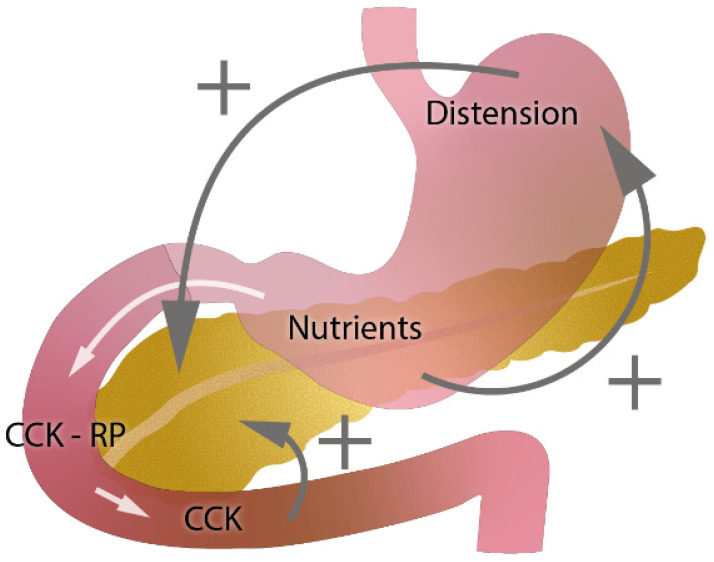
Role of gastric emptying and cholecystokinin (CKK) in exocrine pancreatic secretion (edited according to [4] and developed in collaboration with the Center for E-Learning Service, Faculty of Informatics at Masaryk University).

In gastric resections, asynchrony between gastric emptying and discharge of bile and pancreatic enzymes into the small intestine (postcibal pancreatic asynchrony) and the decreased endogenous stimulation of cholecystokinin CKK [2,4,26] might have a significant impact on pathophysiology (Figure 5, edited according to [4]).

Friess et al. presented a study involving 15 patients after total gastrectomy, all of whom developed severe EPI [105]. Similarly, steatorrhea developed after partial gastrectomy in all subjects, as described in another study involving 30 patients [106].

In another study, Gullo et al. compared pancreatic secretion in patients after total gastrectomy with controls. Lower pancreatic secretion was measured significantly in all patients, and 67% developed steatorrhea [1,107].

The vagus nerve also plays an important role in the regulation of pancreatic secretion through the vagovagal reflex. During extensive gastric surgery, damage to the vagus nerve can result in the development of postoperative EPI without affecting the pancreas itself [1,108]. In two studies evaluating patients after vagotomy, there was a decrease in all exocrine pancreatic enzymes [1,109,110].

CKK is secreted in the upper portion of the duodenum, and therefore, duodenal resection affects pancreatic secretion. In 2019, Beger et al. presented a study comparing pancreatoduodenectomy and duodenum-preserving (DP) resection in patients with chronic pancreatitis. They observed shorter overall hospitalizations and better pancreatic function in patients with DP resections [26,111].

Hudy et al. presented a study suggesting that EPI can develop after esophageal resections. They measured FE-1 in 86 patients, and 16% of patients had developed severe EPI with clinical consequences. All underwent PERT and 90% of the patients displayed symptomatic improvement [112].

After gastrointestinal surgery, every patient is unique and presents different degrees and mechanisms of EPI, so PERT should follow an individual dosage according to the symptoms of the patient. In patients with clinical symptoms such as steatorrhea, weight loss, or other symptoms related to maldigestion, the dosage should be 40,000–50,000 PhU units of lipase per main meal and 20,000–25,000 per snack. PERT can be considered even in the case of asymptomatic patients with fat excretion > 15 g/day because there is a higher risk of developing nutritional deficit later [4,5,26,113].

Core tips: Significant EPI can occur in patients with gastric resections, small intestinal resections, and even in esophageal resections, with GI asynchrony and decreased endogenous stimulation being the main pathophysiological factors. Each patient after gastrointestinal surgery is unique, however, and PERT should be considered in cases with symptoms such as steatorrhea and weight loss.

### 3.5. Zollinger–Ellison Syndrome

Zollinger–Ellison syndrome (ZES) is an endocrinopathy caused by gastrin-secreting tumors responsible for the formation of multiple, refractory, and recurrent peptic ulcers localized in the distal duodenum and proximal jejunum and less commonly in the stomach [114]. ZES is sporadic in 62–80% of cases, and in 20–38% of cases it is associated with multiple endocrine neoplasia type 1 (MEN 1) [115]. It is characterized pathophysiologically by significant hypergastrinemia derived from a gastrin-secreting neuroendocrine tumor with a primary location in the pancreas or duodenum (gastrinoma triangle). Diagnosis is based on the finding of elevated levels of fasting serum gastrin associated with gastric acid hypersecretion and the patient’s history, which is typically characterized by recurrent episodes of peptic ulcer disease or severe reflux esophagitis and/or diarrhea or acid-related symptoms that do not respond to standard treatment regimens [116].

Treatment is based upon the control of gastric acid hypersecretion and of the malignant tumor (gastrinoma) and its possible metastases. PPIs are the most effective antisecretory drugs and can be administered in high doses without drug-related adverse effects. All sporadic localized gastrinomas should be excised or resected if possible [115].

ZES may be a cause leading to EPI. In EPI, the quantity and/or activity of pancreatic digestive enzymes are below the levels required for normal digestion, leading to maldigestion and malabsorption. This may initially present with nonspecific symptoms, such as bloating, abdominal discomfort, steatorrhea, diarrhea, excess flatulence, and weight loss [117]. EPI in the case of ZES is caused by massive acid hypersecretion, with the resulting decreased pH leading to acid inactivation of pancreatic enzymes in the upper small intestine. The optimal activity of pancreatic lipase is at a pH of 8. Because of acid hypersecretion, pancreatic lipase is inactivated, and low pH also renders some primary bile acids insoluble, thereby reducing the formation of micelles needed for the absorption of fatty acids and monoglycerides. This results in the development of EPI, usually manifesting with steatorrhea, which is typical for 5–10% of patients with ZES [2,118,119]. The diagnosis of EPI is best established by tests that directly measure digestion, such as fecal fat quantification. The treatment of EPI relies upon eliminating risk factors for disease progression and providing PERT in those symptomatic patients with low fecal elastase. The goals of this treatment are to normalize digestion, alleviate EPI-related symptoms, and prevent malnutrition [3].

Core tips: ZES is characterized by significant hypergastrinemia derived from a gastrin-secreting neuroendocrine tumor. ZES may be a cause of EPI, as massive acid hypersecretion and decreased pH lead to acid inactivation of pancreatic enzymes in the upper small intestine. This results in the development of EPI, usually manifested with steatorrhea, which is typical for 5–10% of patients with ZES.

### 3.6. Non-Alcoholic Fatty Pancreas Disease

NAFPD is characterized by an increased accumulation of fat in the pancreas, most frequently due to obesity and metabolic syndrome [120]. Whether NAFPD may have a direct role in EPI remains unknown. To date, the data are rather casuistic in origin and include a few reports of patients with steatorrhea and weight loss and findings of fatty infiltration of the pancreas on imaging [121]. The significance of NAFPD in the etiology of EPI is more indirect, as it occurs through its relationship with diabetes mellitus and pancreatic adenocarcinoma. The relationship between NAFPD and diabetes mellitus has been clearly confirmed. NAFPD may be associated with dysfunction and a decrease in beta cells, lipotoxicity, insulin resistance, and inflammation [65,67].

NAFPD has been diagnosed in higher numbers among patients with pancreatic cancer. Chronic inflammation is speculated to be a pathophysiological mechanism, similar to the process of cancerogenesis in nonalcoholic fatty liver disease [122,123].

Core tips: NAFPD may be associated with chronic inflammation, increasing the risk of pancreatic tissue damage and leading to diabetes mellitus, pancreatic adenocarcinoma, and/or EPI. To date, however, the data are rather casuistic in origin.

### 3.7. Age

With improvements in hygiene and health care, life expectancy is increasing. However, elderly patients are at risk of malnutrition caused by the involution of the GI tract’s physiological capacity. Age-related changes in morphology, volume, and function are well known in secretory organs such as the liver, kidney, and intestine [124]. It is obvious that we can expect similar changes in the pancreas.

According to some authors, the volume of the pancreas increases linearly up to the third decade of life and then decreases linearly [125]. Others maintain that it enlarges linearly during childhood and young adulthood, reaches a plateau at 20–60 years, and declines in size thereafter [126]. Not only the pancreatic volume but also the structure of the pancreas transforms with age. An age-related dilatation of the main pancreatic duct has been confirmed in several radiological studies using different imaging techniques [127,128,129,130]. Other well-documented changes related to aging in the pancreas include an increase in the prevalence of cystic lesions observed in both sexes and of calcifications only in men [129], marked lobulation of the pancreas, and fatty replacement of the pancreatic parenchyma in elderly individuals [131,132]. These changes begin to be more prominent during the fifth decade [133]. Semiquantitative elastography has shown that the pancreas becomes stiffer with age [134]. This corresponds to increasing fibrosis, as described histologically [124]. An age-linked decline in parenchymal perfusion of the pancreas [135] may be related to atherosclerosis of the smaller vessels [136]. Interestingly, patients taking angiotensin-converting enzyme (ACE) inhibitors experience pancreatic exocrine insufficiency less frequently [137], presumably due to the potential anti-fibrotic effects of the ACE inhibitors [138].

As a result of these changes, the exocrine function of the pancreas is weakened in healthy elderly people even without any GIT disease. Several studies based upon the secretin–pancreozymin test document the reduction of secretory volume, bicarbonate output, and enzyme output in elderly individuals [139]. The decrease in secretory capacity for chymotrypsin, lipase, and bicarbonate starts in the third decade [125]. Mild EPI (defined by elastase level < 200 μg/g stool) and severe EPI (a fecal elastase level < 100 μg/g) have been observed in 10% and 5%, respectively, of individuals older than 70 years [140].

Impairment of pancreatic functions in the elderly can manifest in a broad spectrum of symptoms, such as maldigestion and malnutrition with steatorrhea, diarrhea, abdominal pain, and weight loss, although patients could be oligosymptomatic due to the unconscious avoidance of certain foods [124]. Elderly individuals with proven EPI should be treated according to recent guidelines for chronic pancreatitis [5]. This could contribute to healthy aging.

Core tips: Aging is related to changes in volume, structure, and secretory capacity of the pancreas. These changes begin to be more prominent during the fifth decade of life. Mild EPI and severe EPI have been observed in 10% and 5%, respectively, of individuals older than 70 years. EPI can manifest as steatorrhea, diarrhea, abdominal pain, and weight loss, although patients could be oligosymptomatic. Elderly individuals with proven EPI should be treated according to recent guidelines for chronic pancreatitis, and this could contribute to healthy aging.

### 3.8. Relationship between Exocrine Pancreatic Secretion and Intestinal Microbiota, Small Intestinal Bacterial Overgrowth (SIBO)

The association between intestinal microflora and general inflammatory, metabolic, and tumor diseases has been confirmed numerous times. Changes in intestinal microflora are also described in patients with chronic pancreatitis [141].

Furthermore, it now seems that there is also a relationship between the intestinal microflora and exocrine pancreatic secretion. The first population-based study published by Frost et al. [142] monitored 1795 people who presented no evidence of any pancreatic disease in their personal histories and each of whom provided a stool sample that was subjected to a 16S ribosomal genetic sequencing analysis and a determination of the levels of fecal elastase-1. The subjects also underwent a secretin stimulation test in order to determine the fluid portion of pancreatic function. This study showed that changes in exocrine pancreatic secretion, evaluated according to elastase-1 levels in the stool, were statistically significantly correlated with changes in intestinal microflora, with people having low levels of elastase-1 showing a significant increase in *Prevotella* spp. and a significant decrease in *Bacteroides* spp. The changes in pancreatic fluid secretion (and thus a function of the pancreatic ductal cells) were also significant, although to a lesser extent. The study confirmed a relationship between exocrine pancreatic secretion and changes in intestinal microflora. Changes detected in the function of pancreatic acini were more significantly pronounced than were the changes in the function of pancreatic ductal cells. On the basis of these results, it is evident that the intestinal microbiome influences exocrine pancreatic secretion.

The increase of the bacterial species *Prevotella* observed in individuals with decreased levels of elastase-1 in their stool is an important factor in the induction of chronic inflammation [143]. Furthermore, *Prevotella* is a producer of hydrogen sulfide, which is known to act as an inductor of pancreatic damage via apoptosis [144].

Small intestinal bacterial overgrowth (SIBO) occurs when there is an abnormal increase in the overall bacterial population in the small intestine, and particularly of bacteria types not commonly found in that part of the GI tract. The relationship between SIBO and EPI seems to be bilateral, and their symptoms largely overlap. EPI unequivocally leads to SIBO, which is present in 15–37% of ChP patients [141,145,146]. Conversely, gut dysbiosis such as SIBO is hypothesized to mediate chronic proinflammatory changes in the pancreas and, hence, SIBO can exacerbate EPI [147]. Therefore, screening for and treatment of SIBO would be beneficial for patients with EPI by breaking the vicious circle of EPI and SIBO potentiation. There are data showing that properly maintained PERT can lead to normalization of the gut microbiome [148].

Core tips: Changes in intestinal microflora have been described in many diseases, including chronic pancreatitis. Furthermore, changes in intestinal microflora have been proven to correlate with changes in exocrine pancreatic secretion and, therefore, the intestinal microbiome evidently influences exocrine pancreatic secretion. The relationship between SIBO and EPI seems to be bilateral, and their symptoms largely overlap. EPI unequivocally leads to SIBO. Conversely, SIBO can exacerbate EPI. Therefore, screening for and treatment of SIBO can be recommended for patients with EPI. Moreover, properly maintained PERT can lead to the normalization of the gut microbiome.

## 4. Conclusions

There are additional causes of EPI beyond just chronic pancreatitis. These causes can be divided into loss of pancreatic parenchyma, inhibition or inactivation of pancreatic secretion, and postcibal pancreatic asynchrony. Furthermore, the etiology of EPI can be divided into diseases having obvious association with EPI and diseases and conditions where the exact mechanisms responsible for EPI have not been elucidated. EPI occurs in a majority of patients with either resectable or unresectable pancreatic cancer, leading to weight loss that correlates with reduced survival. PERT in PDAC patients contributes to an increase in median survival. EPI is also an underestimated complication of pancreatic surgery, and PERT should become an integral part of therapy for patients after pancreatic resections. Significant EPI can also occur in patients with gastric resections, small intestinal resections, and even esophageal resections. Almost 90% of patients with CF present with EPI, and PERT is a cornerstone in the treatment of EPI in CF patients. All types of diabetes are associated with different degrees of EPI. Therefore, diabetics should be screened and treated for EPI. Patients with celiac disease on a gluten-free diet with persistent diarrhea and low fecal elastase levels should receive PERT. Although pancreatic insufficiency seems to be a rather frequently arising extraintestinal manifestation of IBD, its clinical significance remains undefined. EPI is also connected to ZES, NAFPD, and sometimes as a consequence of aging. EPI unequivocally leads to SIBO. Conversely, SIBO can exacerbate EPI. Therefore, screening for and treatment of SIBO can be recommended for patients with EPI. Moreover, properly maintained PERT can lead to the normalization of the gut microbiome.

In summary, clinicians should carefully consider these less-frequently occurring etiologies that can cause malabsorption and maldigestion. PERT should be given to all patients with clinical symptoms of EPI or malabsorption in accordance with the guidelines for chronic pancreatitis.

## Figures and Tables

**Table 1:** Etiologies of exocrine pancreatic insufficiency (adapted from [1,3]).

**Obvious association with EPI**
Chronic pancreatitis (most common)
Pancreatic cancer
Pancreatic resections
Obstruction of the pancreatic duct (periampullary tumors)
Cystic fibrosis
Rare inherited syndromes (Shwachman–Diamond syndrome, etc.)
**Not fully clarified mechanisms associated with EPI**
Diabetes mellitus
Celiac disease
Inflammatory bowel disease (Crohn’s disease, ulcerative colitis)
Gastrointestinal surgeries other than pancreatic resection (e.g., gastric resection)
Zollinger–Ellison syndrome
Nonalcoholic fatty pancreas disease
Age
Composition and diversity of the intestinal microbiome, SIBO

**Table 2:** Etiologies of exocrine pancreatic insufficiency in childhood [53].

Cystic fibrosis (most common)
Shwachman–Diamond syndrome
Chronic pancreatitis
Johanson–Blizzard syndrome
Pearson syndrome
Jeune syndrome
Pancreatic hypoplasia
Pancreatic aplasia
Isolated enzyme deficiencies

## Data Availability

None.
